# Single-Nucleotide Polymorphisms in Genes Associated with Mitochondrial and DNA Damage Response Modulate the Risk of Non-Alcoholic Fatty Liver Disease in Humans

**DOI:** 10.3390/ijms27114854

**Published:** 2026-05-28

**Authors:** Sylwia Ziółkowska, Marcin Kosmalski, Łukasz Kołodziej, Kinga Jarmusz, Magdalena Ejsmont, Tadeusz Pietras, Aleksandra Jabłkowska, Maciej Jabłkowski, Janusz Szemraj, Piotr Czarny

**Affiliations:** 1 Department of Medical Biochemistry, Medical University of Lodz, 92-215 Lodz, Poland; sylwia.ziolkowska@umed.lodz.pl (S.Z.); kinga.jarmusz@student.umed.lodz.pl (K.J.); magdalena.ejsmont@student.umed.lodz.pl (M.E.); janusz.szemraj@umed.lodz.pl (J.S.); 2 Department of Clinical Pharmacology, Medical University of Lodz, 90-153 Lodz, Poland; marcin.kosmalski@umed.lodz.pl (M.K.); tadeusz.pietras@umed.lodz.pl (T.P.); 3 Department of Molecular Genetics, Faculty of Biology and Environmental Protection, University of Lodz, 90-236 Lodz, Poland; lukasz.kolodziej@edu.uni.lodz.pl; 4 Bio-Med-Chem Doctoral School of the University of Lodz and Lodz Institutes of the Polish Academy of Sciences, 90-237 Lodz, Poland; 5 Department of Infectious and Liver Diseases, Medical University of Lodz, 91-347 Lodz, Poland; jablkowska@pro.onet.pl (A.J.); maciej.jablkowski@umed.lodz.pl (M.J.)

**Keywords:** steatosis, SNP genotyping, DNA repair, mitochondrial DNA, mitochondrial DNA degradation

## Abstract

Non-alcoholic fatty liver disease (NAFLD) is one of the most common chronic liver disorders and has been linked to oxidative stress. Therefore, it can be hypothesized that NAFLD may be associated with genes encoding proteins involved in the base-excision repair (BER) pathway. Moreover, mitochondrial dysfunction plays a significant role in the development of NAFLD. In light of these observations, we suggested that fatty liver may be associated with genes that encode proteins responsible for mitochondrial DNA (mtDNA) degradation. This study evaluates single-nucleotide polymorphisms (SNPs) within the *EXOG*, *ENDOG*, *POLG*, *FEN1*, *PARP1*, and *XRCC1* genes in 99 patients and 104 controls. SNP genotyping was performed using TaqMan probes and the findings were presented as odds ratios with corresponding 95% confidence intervals. Each of the eight investigated SNPs was found to modulate the risk of NAFLD occurrence. The analysis revealed that the studied haplotypes of *EXOG* and *XRCC1* significantly affected the frequency of NAFLD in patients. The findings allow us to assume that there is a link between *FEN1*, *PARP1*, *XRCC1*, *POLG*, *EXOG*, and *ENDOG* and liver steatosis. We believe that the impaired repair and degradation of damaged mtDNA may have a significant impact on the development of NAFLD.

## 1. Introduction

In light of the vast global obesity epidemic, particularly in developed countries, research often focuses on understanding its associated diseases. Non-alcoholic fatty liver disease (NAFLD) is estimated to affect one in four people worldwide, increasing the disease awareness in the general population. According to US guidelines for NAFLD management, NAFLD can be defined by the presence of steatosis in at least 5% of hepatocytes confirmed by imaging or histology [1]. The diagnostic criteria for the disease include not only the lack of excessive alcohol consumption but also the exclusion of other causes of hepatic steatosis, such as drugs or hereditary disorders. NAFLD can manifest as non-alcoholic fatty liver (NAFL), a mild form of the disease that can progress to non-alcoholic steatohepatitis (NASH). This form is characterized by the presence of lobular inflammation and hepatocyte ballooning, which can lead to fibrosis and cirrhosis [2]. Currently, there is an ongoing debate about the nomenclature of fatty liver disease. It has been suggested that the term metabolic dysfunction-associated fatty liver disease (MAFLD) places greater emphasis on the metabolic aspect of the disease, which appears to be an inseparable element of its pathogenesis. The diagnostic criteria vary depending on the chosen terminology. NAFLD diagnosis requires the presence of hepatic steatosis, while MAFLD additionally requires obesity, overweight, type 2 diabetes mellitus (T2DM), or, in normal-weight individuals, at least two metabolic risk factors [3,4]. Nevertheless, this article will focus on the term NAFLD, as the patients in this study were diagnosed based on NAFLD criteria.

While the exact etiology of NAFLD remains unclear, a multifactorial interplay of genetic, metabolic, and environmental factors contributes to its pathogenesis [5]. The cornerstone of steatosis is insulin resistance (IR), which impairs glucose uptake by peripheral tissues, leading to increased hepatic glucose production and de novo lipogenesis [6]. Apart from a dysregulation of glucose and lipid metabolism, there are other key molecular mechanisms involved in steatosis: endoplasmic reticulum (ER) stress, inflammation, fibrogenesis, mitochondrial dysfunction, and oxidative stress [7]. The last two mechanisms and their interplay warrant further investigation. Increased reactive oxygen species (ROS) generation overwhelms the antioxidant defense system, leading to lipid peroxidation and protein damage as well as DNA lesion occurrence [8]. The latter may lead to a variety of modifications of the DNA helix, including base modifications, single-strand breaks (SSBs), and double-strand breaks (DSBs). Among these, base modifications are the most common types of DNA damage caused by oxidative stress. Base-excision repair (BER) is a crucial DNA repair pathway for handling these lesions. It is a highly conserved and essential DNA repair mechanism that processes lesions resulting from oxidative damage, alkylation, deamination, or spontaneous base loss [9]. The process starts with the recognition and removal of the lesion. This generates an abasic site, which is subsequently filled in. Any flap DNA fragments are removed, and finally, the separate DNA segments are sealed through ligation, restoring the integrity of the strand [10].

Another factor associated with NAFLD is mitochondrial dysfunction, which impairs hepatocellular β-oxidation of fatty acids, and consequently promotes triglyceride accumulation within the liver. Therefore, this dysfunction reduces ATP production, disrupting the energy balance required for proper liver functioning [11]. Moreover, damaged mitochondria also produce excessive ROS, causing oxidative stress that further harms biological molecules. This creates a damaging feedback loop in which oxidative stress exacerbates mitochondrial impairment. The resulting oxidative damage triggers inflammation and cell death, driving the progression from simple fat accumulation to more severe liver disease. In steatotic livers, antioxidant protection is often insufficient, amplifying this harmful cycle [12].

Taken together, these observations suggest that the accumulation of DNA damage appears to be closely linked to the development of NAFLD. Given the key role of DNA repair and degradation mechanisms in maintaining genomic integrity, we hypothesize that insufficient mtDNA repair capacity and impaired mtDNA degradation might contribute to NAFLD pathogenesis. This hypothesis is further strengthened by our recent findings confirming an association between polymorphisms in genes encoding BER pathway proteins and the presence of fatty liver [13]. In the current article, we present the first reported link between variants in nuclear genes related to mitochondrial DNA maintenance and NAFLD, describing the associated risk estimates for each of the eight single-nucleotide polymorphisms (SNPs) variants studied.

## 2. Results

### 2.1. Single-Nucleotide Polymorphisms in Genes Related to BER Modulate the Risk of NAFLD in Insulin-Resistant Patients

The allele distribution of the studied BER-associated genes differs significantly among the studied groups. The results demonstrate that various variants of *FEN1*, *PARP1*, and *XRCC1* have a considerable impact on the risk of NAFLD. The exact findings can be found in Table 1. The risk is presented as odds ratio (Or) values with 95% CI with corresponding *p*-values, and the differences in allele distribution are expressed as χ^2^. The distribution of all genotypes is consistent with Hardy–Weinberg equilibrium.

The post hoc power analysis shows that most SNPs have a high statistical power (>0.80), ranging from 0.87 to 1.00. However, a few statistically insignificant variants exhibit low power (as low as 0.056), reflecting the limited ability to detect associations for these SNPs. These genotypes are marked as “*” in Table 1 and Table 2.

Genotype-phenotype analyses were performed using the available clinical and biochemical parameters (body mass index; high-density lipoprotein; triglycerides; hepatic steatosis index; fatty liver index). No statistically significant differences were observed between genotypes, with the exception of a nominal association between EXOG rs9838614 and triglyceride levels. Detailed results are provided in the Supplementary Data (Tables S1–S8).

### 2.2. Single-Nucleotide Polymorphisms in Genes Responsible for Maintenance of Mitochondrial Genome Integrity Modulate the Risk of NAFLD in Insulin-Resistant Patients

The allele distribution of the tested genes involved in mtDNA replication, repair, and degradation differ between patients with hepatic steatosis and controls. As we can see in Table 2, the studied SNPs in *EXOG*, *ENDOG,* and *POLG* genes modify NAFLD risk. The findings are demonstrated as ORs with 95% CIs for risk, and χ^2^ for differences in allele distribution. All genotypes and alleles are consistent with Hardy–Weinberg equilibrium.

### 2.3. Haplotypes of Single-Nucleotide Polymorphisms in EXOG Modulate the Risk of NAFLD Occurrence

Since SNPs located in the *EXOG* and *XRCC1* genes are statistically significant, we have assumed that their haplotypes may also alter the NAFLD risk. The analysis reveals that the GA haplotype of *EXOG* significantly increases the OR of hepatic steatosis, while the GG haplotype has a protective effect. Moreover, the *XRCC1* haplotypes CG, TG, TA, and CA have been associated with an increased risk of NAFLD, while the GG haplotype may also be linked to disease susceptibility, as it has been observed only in controls. The results are presented in Table 3.

## 3. Discussion

In our previous study, we confirmed that SNPs in genes related to BER are associated with NAFLD occurrence. Specifically, we identified significant correlations between NAFLD risk and polymorphic variants in key BER genes such as *APEX1*, *NEIL1*, and *LIG3* [13]. Notably, BER is a major pathway responsible for repairing oxidative DNA damage not only in the nucleus but also in mitochondria, where it plays a central role in maintaining mitochondrial DNA (mtDNA) integrity under conditions of elevated oxidative stress [14]. This is particularly relevant in NAFLD, a disease characterized by disturbed mitochondrial biogenesis, homeostasis, and progressive mitochondrial dysfunction that further exacerbates disease development [15]. Importantly, mitochondrial BER relies on core molecules as well as on a broader network of proteins involved in DNA processing and degradation. These proteins support mtDNA maintenance, and include endonucleases, scaffold proteins, and enzymes responsible for replication and repair coordination [16,17]. Therefore, disturbances in both canonical BER components and associated mtDNA maintenance pathways may collectively contribute to NAFLD pathogenesis, providing the rationale for investigating mitochondrial DNA repair- and maintenance-related genes in the present study.

The first studied gene was *EXOG*. It is located on chromosome 3 and encodes the endonuclease G-like 1 protein, which plays a critical role in the mitochondrial BER pathway, particularly the LP-BER. EXOG is one of the seven known mitochondrial DNases—alongside ENDOG, FEN1, DNA2, MGME1, APE1, and MRE11—that participate in mtDNA repair, contributing to mitochondrial genome maintenance. This protein exhibits endonuclease activity toward single-stranded DNA and 5′ to 3′ exonuclease activity, which enables it to remove the flap structure generated by DNA polymerase during LP-BER. While EXOG is known primarily for its role in mitochondrial BER, it also appears to cleave mtDNA flaps during repair and help prevent the accumulation of damaged DNA strands. Knockdown of *EXOG* slows mtDNA loss in infection models, suggesting its functional involvement in mtDNA degradation under pathological conditions [18]. Experimental knockdown of *EXOG* led to a significant increase in mtDNA damage, particularly SSBs, resulting in mitochondrial dysfunction and apoptosis [19]. Although other nucleases such as FEN1 and DNA2 can also process the flap structures in mitochondrial LP-BER, their knockdown did not induce similar detrimental effects, indicating a unique and essential role of EXOG in maintaining mitochondrial genome stability [20]. Additionally, EXOG has been proposed to perform the rate-limiting step in mitochondrial BER by hydrolyzing the third phosphodiester bond from the 5′-abasic site, emphasizing its central function in processing DNA damage within mitochondria [21]. Clinically, both studied variants have been associated with an increased risk of the occurrence of major depressive disorder [22]. The evaluated variants, c.*627G>A and c.-188T>G (rs106580 and rs9838614, respectively), have been identified in the 3′ untranslated region (3′-UTR) of the gene. Our study has found that both studied variants of *EXOG* affect NAFLD occurrence. The findings indicate a strong association between variants of *EXOG* SNPs and liver steatosis. However, the relationship is more pronounced for rs1065800 than for rs9838614. For the former SNP, genotype AA (*p* = 0.030) and allele A (*p* < 0.001) increase the risk of NAFLD, while genotype GG and allele G (*p* < 0.001) reduce the risk. In the latter SNP, genotypes TT (*p* = 0.007) and GG (*p* = 0.003) elevate the risk, and the presence of genotype TG has been associated with a decreased frequency of NAFLD in patients (Table 2). Since both SNPs show significant associations in studied *EXOG* SNPs, it is reasonable to assume that their haplotypes are related to the occurrence of NAFLD. Haplotype AT may increase the risk of NAFLD (*p* = 0.014), but haplotype GG lessens the risk of liver steatosis (*p* < 0.001) (Table 3).

The next gene is *ENDOG*, located on chromosome 9, which encodes the endonuclease G protein, a paralogue of EXOG. This nuclear-encoded protein resides in the mitochondrial intermembrane space but can translocate to the nucleus during apoptosis. ENDOG is an endonuclease targeting GC-rich mtDNA regions and is implicated in mtDNA degradation. Under oxidative stress, ENDOG promotes the removal of damaged mtDNA fragments in coordination with compensatory replication [23]. ENDOG, as well as EXOG, is one of the mitochondrial DNases. Beyond its DNA-cleaving function, ENDOG has been implicated in the pathogenesis of mitochondria-related diseases, including cardiac hypertrophy, Parkinson’s disease, and obesity, highlighting its clinical significance [24,25,26]. Moreover, cytoplasmic ENDOG has additional regulatory roles. It can repress mTORC1 signaling, induce autophagy, and activate the mTORC2–AKT–ACLY axis, promoting acetyl-CoA production. Interestingly, ENDOG may also translocate to the ER, interact with BiP, and trigger the unfolded protein response (UPR) by activating IRE1α and PERK, leading to enhanced lipid synthesis. In female mice, these actions collectively alleviate high-fat diet (HFD)-induced NAFLD [27]. Our study has demonstrated the link between fatty liver and ENDOG. The presence of a specific polymorphism, c.-394T>C (rs2977998), which is located upstream of the *ENDOG* gene, has been observed to significantly influence NAFLD occurrence in patients. The risk has been estimated to be higher in the presence of genotype CC and allele C, and lower in the presence of genotype TT and allele T (*p* < 0.001) (Table 2).

The *POLG* gene, located on chromosome 15, encodes the catalytic subunit of DNA polymerase gamma (Pol γ), the key enzyme responsible for mtDNA replication and repair. The fully functional mtDNA polymerase is a heterotrimeric complex, consisting of one *POLG*-encoded catalytic subunit and a homodimer of accessory subunits encoded by the *POLG2* gene located on chromosome 17. POLG is capable of performing both short- and long-patch BER [28]. Besides its role in maintaining mitochondrial genome integrity through DNA repair, the protein is also involved in the degradation of damaged mtDNA. The process is primarily carried out by components of the mitochondrial replication machinery, with POLG as the crucial player. Apart from its polymerization activity, POLG possesses 3′ to 5′ exonuclease proofreading capability, which enables the degradation of damaged mtDNA fragments. Under conditions of stress or damage, POLG switches from DNA synthesis to exonucleolytic activity, removing damaged DNA ends. This is complemented by other mitochondrial nucleases, such as EXOG, and by helicases that assist in processing damaged mtDNA [29,30]. Mutations and reduced activity of POLG have been associated with neurological diseases characterized by mtDNA depletion, deletions, or accumulation of mutated mtDNA, highlighting its critical role in mitochondrial function [30,31]. A *POLG* variant, p.Gln1236His, is associated with altered mtDNA copy number in NAFLD and may contribute to mitochondrial dysfunction, disease progression, and correlate with fibrosis severity [32]. Nevertheless, in our study, another *POLG* variant, c.-1370T>A (rs1054875), has been noted to influence NAFLD occurrence. Genotype AA (*p* = 0.002) and allele A (*p* = 0.027) increase disease risk, but genotype AT (*p* = 0.022) and allele T (*p* = 0.046) have the opposite effect (Table 2).

*FEN1* encodes a flap endonuclease crucial for mitochondrial LP-BER, replication fork resolution, and Okazaki fragment maturation. The protein is associated with altered lipid profiles, including HDL, total cholesterol, and triglycerides, as well as lower serum omega-3 levels in individuals with metabolic syndrome [33,34]. Moreover, a meta-analysis of 20 studies including 7366 cases and 9028 controls has shown a decreased risk of cancer in individuals carrying this polymorphism [35]. The c.-441G>A (rs174538) SNP in *FEN1* has been analyzed in this study. We noticed that genotype AA increases the risk of NAFLD (*p* = 0.020), as does genotype AG (*p* < 0.001) and allele A. Simultaneously, genotype GG and allele G both reduce the risk (*p* < 0.001) (Table 1).

*PARP1* encodes a multifunctional enzyme (poly(ADP-ribose) polymerase 1) that plays a central role in cellular responses to DNA damage, particularly through the BER pathway and homologous recombination in the repair of DSBs. Beyond its repair function, PARP1 is also involved in regulating transcription, apoptosis, and inflammatory responses [36,37]. PARP1 is activated in response to oxidative stress, a key mechanism underlying lipotoxicity, where it contributes to DNA damage signaling and activates the ERK pathway. This activation has been observed under HFD conditions, linking PARP1 activity with metabolic stress and de novo lipogenesis, which positions it as a potential contributor to the development and progression of NAFLD [38]. Different *PARP1* SNPs have been correlated with both decreased and increased risk of several cancers, including gallbladder cancer, esophageal cancer in smokers, gastric cancer, thyroid cancer, cervical cancer, brain cancer, and epithelial ovarian cancer [39,40,41,42,43,44,45,46]. In our study of the c.2285T>C (rs1136410) variant, we have observed an elevated risk of liver steatosis in the presence of genotype AA and allele A, and reduced risk with allele G (*p* < 0.001) (Table 1).

The *XRCC1* gene, located on chromosome 19, encodes the X-ray repair cross-complementing protein 1, which plays a crucial role in multiple DNA repair pathways, including BER, SSB repair, and non-homologous end joining. In the BER pathway, XRCC1 functions as a scaffold protein that coordinates and stabilizes the activity of various DNA repair enzymes, ensuring the efficiency and fidelity of the repair process [47,48]. The studied polymorphism, c.580C>T (rs1799782), which causes an arginine-to-tryptophan substitution at codon 194, is associated with an increased susceptibility to hepatocellular carcinoma in both Asian and Caucasian populations [49]. Another variant, c.1196A>G (rs25487), causes a glutamine-to-arginine substitution at codon 399 and is located in the breast cancer 1 C terminus domain, which is responsible for interactions with PARP1 [50]. This variant has also been linked to cancer susceptibility, including hepatocarcinoma, which suggests a potential role in the pathophysiological processes occurring in liver tissue chronically exposed to damage, as observed in NAFLD [51,52,53]. In our study of c.580C>T (rs1799782), the occurrence of genotype AA and allele A significantly increases the risk of NAFLD, while genotype GG and allele G reduce it. In c.1196A>G (rs25487) genotypes CC and CT and allele C increase the OR, while genotype TT and allele T decrease the risk (Table 1).

In the present study, the analyzed SNPs are predominantly located in regulatory regions or represent coding variants with potential functional consequences. Variants in regulatory regions may influence gene expression through multiple mechanisms beyond simple sequence variation. Promoter SNPs can alter transcription factor binding affinity and affect transcription initiation, while variants in non-coding regions may also influence chromatin structure and regulatory element activity [54]. Similarly, polymorphisms located in the 3′-UTR may impact post-transcriptional regulation by modifying microRNA (miRNA) binding sites, as well as by affecting mRNA stability, translational efficiency, or interactions with RNA-binding proteins [55,56]. These regulatory effects have been widely described as mechanisms linking non-coding genetic variation to altered gene expression and disease susceptibility [57,58].

Among the analyzed variants, *EXOG* rs1065800 (3′-UTR) and rs9838614 (promoter), as well as *ENDOG* rs2977998 and *POLG* rs1054875 (promoter and enhancer, respectively), may potentially influence transcriptional regulation or mRNA stability. However, direct functional evidence for these SNPs remains limited. In contrast, the *FEN1* rs174538 promoter variant has been previously shown to affect promoter activity, suggesting a possible impact on gene expression [59]. Furthermore, coding polymorphisms such as *PARP1* rs1136410 (Val762Ala) and *XRCC1* rs1799782 (Arg194Trp) and rs25487 (Arg399Gln) have been functionally characterized and are known to influence protein activity, stability, or interactions within the BER pathway [60,61]. In particular, the *PARP1* rs1136410 variant has been associated with reduced enzymatic activity and altered response to DNA damage [60]. In contrast, *XRCC1* polymorphisms, especially rs25487, have been shown to impair DNA repair capacity, likely through disruption of protein–protein interactions within the BER complex, including interactions with PARP1, DNA ligase III, and DNA polymerase β [62,63]. Additionally, both *XRCC1* rs25487 and rs1799782 have been associated with altered DNA repair efficiency in phenotypic and epidemiological studies [61,64].

Other links between DNA repair and fatty liver disease have also been reported, for example, in ataxia–telangiectasia (A–T), a rare genetic disorder primarily caused by mutations in the ATM gene, which plays a critical role in the cellular response to DNA damage, particularly in DSB repair. Recent findings have shown that significant hepatic fibrosis is present in approximately 20% of A–T patients, suggesting that defective DNA repair may contribute to liver pathology [65]. Another example linking NAFLD and mitochondrial dysfunction is a maternal Western-style diet (mWSD), which has been used as a model to induce NAFLD, allowing for the assessment of the impact of maternal diet on the development of liver pathology in the offspring. MWSD in nonhuman primates has been shown to cause early liver changes in offspring, including disrupted gene expression linked to mitochondria dysfunction, oxidative stress, and a reduced antioxidant response, despite normal body weight and liver fat. When combined with a postweaning WSD, these effects intensify, promoting fibrosis, ER stress, and pro-inflammatory metabolic changes [66]. Moreover, upregulation of the Gadd45α protein, which is mainly responsible for cell growth arrest and also stimulates DNA excision repair pathways, is present in NASH models of mice, which may suggest a protective role against steatohepatitis [67,68]. Accordingly, reduced nucleotide-excision repair (NER) activity in obese patients with fatty liver may impair DNA repair capacity, contributing to disease progression [69]. Overall, dysregulation of DNA repair processes may play a key role in NAFLD pathogenesis. These associations underscore the broader impact of genomic instability on liver health and support the hypothesis that compromised DNA repair pathways and mitochondrial dysfunction can promote hepatic steatosis.

It should be emphasized that the development of NAFLD results from a complex interplay between genetic predisposition and environmental factors, including diet, physical activity, alcohol consumption, pharmacotherapy, and other lifestyle-related variables [7]. Therefore, the effect of a given SNP may be modified or masked by environmental exposures, potentially also through epigenetic mechanisms. In the present study, clinical and biochemical data were available only for the NAFLD group. Consequently, genotype-phenotype analyses were performed exclusively within affected patients using parameters such as BMI, HDL, TG, HSI, and FLI. Importantly, most analyses comparisons did not reveal statistically significant differences (Supplementary Data; Tables S1–S8). The only observed association involved TG levels in relation to the EXOG rs9838614 polymorphism (Supplementary Data; Table S2); however, this finding should be interpreted with caution because of the potential influence of confounding factors. Although we did not observed the link between the investigated SNPs and most clinical variables, this does not exclude the possibility of the potential relationship between selected BER pathway variants and the development of steatosis. Notably, all patients included in the study were receiving anti-diabetic treatment, which may have affected biochemical parameters and reduced detectable inter-genotype differences. Importantly, the aim of this study was not to assess whether SNPs modulate severity of the disease, but to explore whether BER-related polymorphisms may be associated with NAFLD occurrence and thereby provide a basis for further mechanistic investigations. Given the established role of oxidative stress in NAFLD and its link to BER pathway activity, our findings should be considered exploratory and hypothesis-generating, providing a rationale for future studies integrating detailed environmental and functional data.

One of the limitations of this study is the lack of functional validation of the identified genetic associations. A substantial proportion of SNPs are located in regulatory regions, where they may influence gene expression through altered transcription factor binding or post-transcriptional mechanisms. Since no gene expression analysis or functional assays have been performed, and the available evidence in the literature is scarce, the impact of the studied variants on DNA repair functionality cannot be directly assessed. Moreover, the cohort has been restricted to patients with T2DM to ensure a relatively homogeneous metabolic background with a high prevalence of IR. However, it must be stated that this may limit generalizability to non-diabetic NAFLD populations. In addition, NAFLD diagnosis in patients involved in the present study was based on ultrasonography and non-invasive indices (HSI and FLI), which do not allow for the assessment of disease severity or fibrosis stage, restricting the analysis to hepatic steatosis. Furthermore, although post hoc power analysis indicates adequate statistical power for most SNPs (>0.80), several variants show limited power, increasing the risk of type II error. Haplotype analyses were not accompanied by formal power calculations due to their multi-allelic nature and low frequency distribution, and should therefore be considered exploratory. Finally, the study population was relatively homogeneous (Caucasian), which may limit generalizability. Replication in larger and more diverse cohorts is required, and the identified variants should currently be considered research markers rather than clinically applicable biomarkers.

## 4. Materials and Methods

### 4.1. Patients and Ethics

Participants were recruited from two Polish hospitals: Norbert Barlicki Memorial Teaching Hospital in Lodz, Poland, and Bieganski Provincial Specialist Hospital in Lodz, Poland. The study group consisted of 99 patients diagnosed with NAFLD, whereas the control group comprised 104 individuals without any signs of fatty liver. Steatosis confirmed by ultrasonography was a prerequisite for inclusion, whereas an age below 18 years and history of cancers or liver diseases were exclusion criteria for the study. Additionally, non-invasive indices of hepatic steatosis, including the hepatic steatosis index (HSI) and fatty liver index (FLI), were calculated to support the assessment of liver fat accumulation. IR was not directly assessed; instead, all participants had a confirmed diagnosis of T2DM, which is strongly associated with its presence. The patients’ characteristics are presented in Table 4, and clinical and biochemical parameters are shown in Table 5. The study was conducted in accordance with the Declaration of Helsinki and approval was obtained from the Bioethics Committee of the Medical University of Lodz, Poland (no. RNN/160/20/KE), and all participants provided written informed consent.

### 4.2. Sample Collection and DNA Isolation

Whole blood samples were collected from each participant to tubes containing EDTA, aliquoted (200 µL), and stored at −20 °C until DNA isolation. Genomic DNA was isolated using the Invisorb^®^ Spin Blood Mini Kit (Invitek Molecular GmbH, Berlin, Germany). DNA concentration and purity were determined by measuring absorbance at 260 nm and 280 nm (Picodrop, Syngen Biotech, Wroclaw, Poland).

### 4.3. SNP Selection

In accordance with data in the public domain of the National Center for Biotechnology Information, the SNP database, available at http://www.ncbi.nlm.nih.gov/snp (Accessed on 15 November 2023; Bethesda, MD, USA), was used to select eight potentially functional SNPs. The polymorphisms are present in genes related to maintaining mitochondrial genome integrity as well as to the BER pathway. The criteria for SNP selection were as follows: (i) localization in regulatory regions or in a coding region that causes a non-synonymous substitution; (ii) a minor allele frequency (MAF) greater than 0.05 in a European population. The studied polymorphisms are presented in Table 6.

### 4.4. SNP Genotyping

SNP genotyping was performed through qPCR using TaqMan™ Universal PCR Master Mix (Applied Biosystems™, Waltham, MA, USA). The TaqMan Assay IDs used for genotyping are presented in Table 6. Reactions were performed in a Bio-Rad CFX96 thermocycler (Bio-Rad Laboratories Inc., Hercules, CA, USA). Results were analyzed using CFX Manager Software Version 3.1 (Bio-Rad Laboratories Inc.).

### 4.5. Statistical Analysis

The collected data were analyzed in SigmaPlot 11.0 (Systat Software Inc., San Jose, CA, USA). The odds ratio (OR) and its corresponding 95% confidence interval (95% CI) were calculated to estimate NAFLD risk. To evaluate the differences between distributions of alleles and genotypes in studied groups, we performed chi-square (χ^2^) analysis. Furthermore, the haplotype analysis was assessed based on the studied genotypes of four SNPs, and SHEsisPlus software (http://shesisplus.bio-x.cn/SHEsis.html, accessed on 20 June 2024) was used as an online tool [70]. Haplotypes with a frequency < 0.03 were excluded from the analysis. A post hoc power analysis was performed using G*Power (version 3.1.9.7.) [71] based on the observed sample size, allele frequencies, odds ratios, and a significance level of 0.05. Logistic regression was used as the underlying statistical framework for power estimation. Power was calculated for individual SNPs to evaluate the adequacy of the study sample size in detecting the observed genetic effects. Due to the multi-allelic structure and low frequencies of haplotypes, formal power calculations were not performed for haplotype-based analyses. Genotype-phenotype associations were analyzed using the Kruskal-Wallis test. Normality of distribution was assessed using the Shapiro-Wilk test. In cases where one genotype group was not represented in the study population, genotype-phenotype comparisons were performed using the Mann-Whitney U test instead of the Kruskal-Wallis test.

## Figures and Tables

**Table 1:** Association between the studied single-nucleotide polymorphism and NAFLD. The table presents the distribution of genotypes and alleles of *FEN1* rs174538; *PARP1* rs1136410; and *XRCC1* rs1799782 and rs25487 as well as ORs with 95% CIs in groups of patients with NAFLD and controls.

Genotype/Allele	NAFLD (*n* = 99)		Control (*n* = 104)		Crude OR (95% CI)	*p*-Value
	Number	Frequency	Number	Frequency		
*FEN1* rs174538						
AA	48	0.485	6	0.058	15.373 (6.165–38.331)	<0.001
AG	40	0.404	26	0.250	2.034 (1.118–3.700)	0.020
GG	11	0.111	72	0.692	0.056 (0.026–0.118)	<0.001
χ^2^ = 80.393; *p* ≤ 0.001						
A	136	0.687	38	0.183	7.706 (4.530–13.107)	<0.001
G	62	0.313	170	0.817	0.130 (0.076–0.221)	<0.001
*PARP1* rs1136410						
AA	73	0.737	27	0.260	8.007 (4.279–14.982)	<0.001
AG	26	0.263	28	0.269	0.967 (0.518–1.802)	0.915 *
GG	0	0.000	49	0.471	<0.001 (0.000- +inf)	0.993 *
χ^2^ = 70.153; *p* ≤ 0.001						
A	172	0.869	82	0.394	6.319 (3.817–10.462)	<0.001
G	26	0.131	126	0.606	0.158 (0.096–0.262)	<0.001
*XRCC1* rs1799782						
AA	21	0.212	1	0.010	27.731 (3.651–210.626)	0.001
AG	9	0.091	15	0.144	0.593 (0.247–1.426)	0.243
GG	69	0.697	88	0.846	0.418 (0.211–0.829)	0.012
χ^2^ = 21.871; *p* ≤ 0.001						
A	51	0.258	17	0.082	2.439 (1.490–3.991)	<0.001
G	147	0.742	191	0.918	0.410 (0.251–0.671)	<0.001
*XRCC1* rs25487						
CC	39	0.394	22	0.212	2.258 (1.220–4.179)	0.010
CT	46	0.465	30	0.288	2.212 (1.238–3.951)	0.007
TT	14	0.141	52	0.500	0.170 (0.086–0.336)	<0.001
χ^2^ = 29.880; *p* ≤ 0.001						
C	124	0.626	74	0.356	2.429 (1.656–3.562)	<0.001
T	74	0.374	134	0.644	0.412 (0.281–0.604)	<0.001

χ^2^—chi-square; CI—confidence interval; OR—odds ratio; *—power analysis < 0.8.

**Table 2:** Association between the studied single-nucleotide polymorphism and NAFLD. The table presents the distribution of genotypes and alleles of *EXOG* rs1065800 and rs9838614; *ENDOG* rs2977998; and *POLG* rs1054875, as well as ORs with 95% CIs in groups of patients with NAFLD and controls.

Genotype/Allele	NAFLD (*n* = 99)		Control (*n* = 104)		Crude OR (95% CI)	*p*-Value
	Number	Frequency	Number	Frequency		
*EXOG* rs1065800						
AA	18	0.182	8	0.077	2.667 (1.102–6.453)	0.030
AG	79	0.798	74	0.712	1.601 (0.837–3.063)	0.155
GG	2	0.020	22	0.212	0.077 (0.018–0.337)	<0.001
χ^2^ = 20.566; *p* ≤ 0.001						
A	115	0.581	90	0.433	4.016 (2.007–8.036)	<0.001
G	83	0.419	118	0.567	0.249 (0.124–0.498)	<0.001
*EXOG* rs9838614						
TT	16	0.162	4	0.038	4.819 (1.551–14.973)	0.007
TG	67	0.677	98	0.942	0.128 (0.051–0.323)	<0.001
GG	16	0.162	2	0.019	9.831 (2.197–43.984)	0.003
χ^2^ = 23.804; *p* ≤ 0.001						
T	99	0.500	106	0.510	0.902 (0.477–1.706)	0.752 *
G	99	0.500	102	0.490	1.108 (0.586–2.096)	0.752 *
*ENDOG* rs2977998						
CC	56	0.566	29	0.279	3.368 (1.877–6.043)	<0.001
CT	38	0.384	39	0.375	1.038 (0.589–1.831	0.897 *
TT	5	0.051	36	0.346	0.100 (0.038–0.269)	<0.001
χ^2^ = 31.925; *p* ≤ 0.001						
C	150	0.758	97	0.466	3.090 (2.019–4.730)	<0.001
T	48	0.242	111	0.534	0.324 (0.211–0.495)	<0.001
*POLG* rs1054875						
AA	28	0.283	11	0.107	3.334 (1.555–7.149)	0.002
AT	52	0.525	71	0.689	0.514 (0.291–0.910)	0.022
TT	19	0.192	21	0.204	0.939 (0.470–1.876)	0.858
χ^2^ = 10.370; *p* = 0.006						
A	108	0.545	93	0.451	1.665 (1.059–2.619)	0.027
T	90	0.455	113	0.549	0.632 (0.403–0.992)	0.046

χ^2^—chi-square; CI—confidence interval; OR—odds ratio; *—power analysis < 0.8.

**Table 3:** Haplotypes of single-nucleotide polymorphisms in *EXOG* and *XRCC1* modulate the risk of NAFLD.

Haplotype	NAFLD (*n* = 99)		Control (*n* = 104)		Crude OR (95% CI)	*p*-Value
	Number	Frequency	Number	Frequency		
*EXOG rs9838614 and rs1065800*						
TA	81	0.409	78	0.375	1.153 (0.774–1.719)	0.541
TG	18	0.090	24	0.115	0.766 (0.402–1.46)	0.514
GG	65	0.328	94	0.451	0.592 (0.396–0.887)	0.011
GA	34	0.171	12	0.057	3.386 (1.698–6.751)	0.003
*XRCC1 rs25487 and rs1799782*						
CG	90	0.454	58	0.278	2.155 (1.426–3.255)	0.003
TG	57	0.287	25	0.120	2.959 (1.761–4.972)	<0.001
TA	17	0.085	1	0.004	19.441 (2.561–147.539)	<0.001
CA	34	0.171	4	0.019	10.573 (3.676–30.403)	<0.001
GG	0	0	108	0.519	N/A	<0.001

CI—confidence interval; N/A—not applicable; OR—odds ratio.

**Table 4:** The characteristics of patients which were included in the study.

Number of Patients (Male/Female)	47/52
Mean age of patients ± SD	64.02 ± 8.06
Mean BMI of patients ± SD	33.24 ± 5.30

BMI—body mass index; SD—standard deviation.

**Table 5:** Clinical and biochemical features of patients with non-alcoholic fatty liver disease.

Parameters	Mean	SD
Age, years	64.02	8.06
BMI, kg m^−2^	33.24	5.30
Fasting glucose, mg dL^−1^	129.85	24.91
HbA1c, %	7.20	1.44
ALT, U L^−1^	35.18	21.58
AST, U L^−1^	29.70	14.07
Total cholesterol, mg dL^−1^	185.14	47.77
HDL cholesterol, mg dL^−1^	52.94	15.94
LDL cholesterol, mg dL^−1^	101.71	38.91
TG, mg dL^−1^	173.61	94.59
HSI	45.96	7.00
FLI	85.52	12.87

ALT—alanine transaminase; AST—aspartate aminotransferase; BMI—body mass index; FLI—fatty liver index; HDL—high-density lipoprotein; HSI—hepatic steatosis index; LDL—low-density lipoprotein; SD—standard deviation; TG—triglycerides.

**Table 6:** Single-nucleotide polymorphisms selected for the study.

Gene	NCBI db SNP ID	SNP Localization	MAF *
*EXOG*	rs1065800	c.*627G>A	0.36548
*EXOG*	rs9838614	c.-188T>G	0.36469
*ENDOG*	rs2977998	c.-394T>C	0.24207
*POLG*	rs1054875	c.-1370T>A	0.31980
*FEN1*	rs174538	c.-441G>A	0.29128
*PARP1*	rs1136410	c.2285T>C	0.15757
*XRCC1*	rs1799782	c.580C>T	0.06264
*XRCC1*	rs25487	c.1196A>G	0.35787

*—minor allele frequency (MAF) in European population.

## Data Availability

Additional data can be requested via e-mail from the corresponding authors.

## Supplementary Materials

The following supporting information can be downloaded at: https://www.mdpi.com/article/10.3390/ijms27114854/s1.

## Abbreviations

The following abbreviations are used in this manuscript:

BER	base-excision repair
BMI	body mass index
CI	confidence interval
DSB	double-strand break
ER	endoplasmic reticulum
FLI	fatty liver index
HFD	high-fat diet
HSI	hepatic steatosis index
IR	insulin resistance
MAF	minor allele frequency
MAFLD	metabolic dysfunction-associated fatty liver disease
mtDNA	mitochondrial DNA
mWSD	maternal Western-style diet
NAFL	non-alcoholic fatty liver
NAFLD	non-alcoholic fatty liver disease
NASH	non-alcoholic steatohepatitis
NER	nucleotide-excision repair
OR	odds ratio
ROS	reactive oxygen species
SD	standard deviation
SNP	single-nucleotide polymorphism
SSB	single-strand break
T2DM	type 2 diabetes mellitus
UPR	unfolded protein response
WSD	Western-style diet
