# Supplementary material for: Single-Nucleotide Polymorphisms in Genes Associated with Mitochondrial and DNA Damage Response Modulate the Risk of Non-Alcoholic Fatty Liver Disease in Humans
Source: Int J Mol Sci. 2026 May 28;27(11):4854. doi: 10.3390/ijms27114854 (PMC13256566; doi:10.3390/ijms27114854)
Supplement: Supplementary file 1 [file ijms-27-04854-s001.zip › ijms-4281748-supplementary.pdf]

Supplementary Table S1. Genotype-phenotype associations for *EXOG* rs1065800 polymorphism in the NAFLD group.

Clinical/biochemical parameter	Genotype	n	Mean ± SD/ Median (IQR)	Test used	p-value
BMI (kg/m²)	AA	18	33.12 (29.53-36.76)	Mann-Whitney	0.6414
	AG	81	32.28 (28.74-36.68)		
	GG	0	-	-	
HDL (mg/dL)	AA	18	47.00 (43.75-61.00)	Mann-Whitney	0.8589
	AG	81	49.00 (44.00-58.00)		
	GG	0	-	-	
Triglycerides (TG)	AA	18	118.50 (109.50-197.25)	Mann-Whitney	0.2563
	AG	81	147.00 (116.00-209.00)		
	GG	0	-	-	
HSI	AA	18	45.00 (39.50-49.75)	Mann-Whitney	0.7274
	AG	81	45.70 (40.85-50.25)		
	GG	0	-	-	
FLI	AA	18	88.94 (76.84-96.58)	Mann-Whitney	0.8608
	AG	81	90.93 (75.35-96.64)		
	GG	0	-	-	

Data are presented as mean ± SD or median (IQR), as appropriate. Normality of distribution was assessed using the Shapiro-Wilk test. Comparisons between genotype groups were performed using Kruskal-Wallis test, or Mann-Whitney U test, depending on number of analyzed groups. Statistical significance was set at $p<0.05$.

Supplementary Table S2. Genotype-phenotype associations for *EXOG* rs9838614 polymorphism in the NAFLD group.

Clinical/biochemical parameter	Genotype	n	Mean ± SD/ Median (IQR)	Test used	p-value
BMI (kg/m²)	GG	16	32.99 (28.52-35.87)	Kruskal-Wallis	0.6964
	GT	67	31.98 (28.73-36.33)		
	TT	16	33.42 (31.71-37.03)		
HDL (mg/dL)	GG	16	46.00 (43.25-62.25)	Kruskal-Wallis	0.6877
	GT	67	49.00 (44.00-60.00)		
	TT	16	50.50 (39.75-52.00)		
Triglycerides (TG)	GG	16	115.00 (105.75-137.00)	Kruskal-Wallis	0.0016
	GT	67	162.00 (132.00-218.00)		
	TT	16	118.00 (93.25-162.00)		
HSI	GG	16	44.90 (38.30-49.48)	Kruskal-Wallis	0.4210
	GT	67	44.20 (40.30-50.90)		
	TT	16	47.35 (43.98-49.50)		
FLI	GG	16	84.31 (74.27-97.42)	Kruskal-Wallis	0.9041
	GT	67	90.93 (75.57-97.38)		
	TT	16	91.43 (74.60-96.04)		

Data are presented as mean ± SD or median (IQR), as appropriate. Normality of distribution was assessed using the Shapiro-Wilk test. Comparisons between genotype groups were performed using Kruskal-Wallis test, or Mann-Whitney U test, depending on number of analyzed groups. Statistical significance was set at $p<0.05$.

Supplementary Table S3. Genotype-phenotype associations for *ENDOG* rs2977998 polymorphism in the NAFLD group.

**Clinical/biochemical parameter**	**Genotype**	**n**	**Mean ± SD/ Median (IQR)**	**Test used**	**p-value**
BMI (kg/m²)	CC	56	33.14 (29.31-37.14)	Kruskal-Wallis	0.2676
	CT	38	32.81 (27.87-36.36)		
	TT	5	27.99 (26.35-35.00)		
HDL (mg/dL)	CC	56	49.00 (44.00-61.5)	Kruskal-Wallis	0.5758
	CT	38	48.00 (43.50-57.00)		
	TT	5	49.00 (43.50-56.50)		
Triglycerides (TG)	CC	56	142.50 (111.75-209.75)	Kruskal-Wallis	0.9114
	CT	38	143.50 (113.00-209.25)		
	TT	5	143.00 (104.00-201.00)		
HSI	CC	56	45.80 (41.45-51.08)	Kruskal-Wallis	0.2219
	CT	38	45.55 (40.20-48.76)		
	TT	5	40.20 (37.30-45.40)		
FLI	CC	56	92.17 (77.04-97.88)	Kruskal-Wallis	0.4800
	CT	38	90.81 (73.24-96.33)		
	TT	5	79.44 (66.78-93.70)		

Data are presented as mean ± SD or median (IQR), as appropriate. Normality of distribution was assessed using the Shapiro-Wilk test. Comparisons between genotype groups were performed using Kruskal-Wallis test, or Mann-Whitney U test, depending on number of analyzed groups. Statistical significance was set at $p<0.05$.

Supplementary Table S4. Genotype-phenotype associations for *POLG* rs1054875 polymorphism in the NAFLD group.

Clinical/biochemical parameter	Genotype	n	Mean ± SD/ Median (IQR)	Test used	p-value
BMI (kg/m²)	AA	28	31.15 (27.50-35.06)	Kruskal-Wallis	0.1695
	AT	53	33.56 (28.73-37.84)		
	TT	18	33.50 (31.72-35.28)		
HDL (mg/dL)	AA	28	49.50 (44.25-63.50)	Kruskal-Wallis	0.2053
	AT	53	48.00 (44.00-57.00)		
	TT	18	47.00 (39.75-56.00)		
Triglycerides (TG)	AA	28	173.50 (120.75-254.75)	Kruskal-Wallis	0.1594
	AT	53	135.00 (102.50-202.00)		
	TT	18	157.50 (109.75-206.25)		
HSI	AA	28	43.30 (40.35-47.88)	Kruskal-Wallis	0.1870
	AT	53	45.90 (39.55-51.15)		
	TT	18	47.30 (42.70-49.38)		
FLI	AA	28	86.89 (76.33-94.41)	Kruskal-Wallis	0.6492
	AT	53	91.96 (73.15-98.07)		
	TT	18	88.63 (77.78-94.83)		

Data are presented as mean ± SD or median (IQR), as appropriate. Normality of distribution was assessed using the Shapiro-Wilk test. Comparisons between genotype groups were performed using Kruskal-Wallis test, or Mann-Whitney U test, depending on number of analyzed groups. Statistical significance was set at $p<0.05$.

Supplementary Table S5. Genotype-phenotype associations for *FEN* rs174538 polymorphism in the NAFLD group.

**Clinical/biochemical parameter**	**Genotype**	**n**	**Mean ± SD/ Median (IQR)**	**Test used**	**p-value**
BMI (kg/m²)	AA	47	32.87 (28.87- 36.33)	Kruskal-Wallis	0.7070
	AG	40	32.28 (28.04- 36.00)		
	GG	12	33.84 (28.88- 37.75)		
HDL (mg/dL)	AA	47	46.00 (42.00-56.00)	Kruskal-Wallis	0.1030
	AG	40	51.00 (46.00-60.00)		
	GG	12	53.50 (40.25-61.50)		
Triglycerides (TG)	AA	47	140.00 (113.00-211.00)	Kruskal-Wallis	0.9077
	AG	40	150.00 (110.75-200.75)		
	GG	12	140.00 (112.00-256.25)		
HSI	AA	47	44.90 (40.30-49.90)	Kruskal-Wallis	0.7316
	AG	40	45.35 (40.95-49.85)		
	GG	12	47.80 (42.05-52.78)		
FLI	AA	47	87.97 (73.86-96.25)	Kruskal-Wallis	0.5500
	AG	40	92.10 (73.24-97.45)		
	GG	12	93.82 (81.41-96.55)		

Data are presented as mean ± SD or median (IQR), as appropriate. Normality of distribution was assessed using the Shapiro-Wilk test. Comparisons between genotype groups were performed using Kruskal-Wallis test, or Mann-Whitney U test, depending on number of analyzed groups. Statistical significance was set at $p<0.05$.

Supplementary Table S6. Genotype-phenotype associations for *PARP1* rs1136410 polymorphism in the NAFLD group.

Clinical/biochemical parameter	Genotype	n	Mean ± SD/ Median (IQR)	Test used	p-value
BMI (kg/m²)	AA	72	32.80 (28.74-36.41)	Mann-Whitney	0.6746
	AG	27	33.14 (28.85-37.18)		
	GG	0	-	-	
HDL (mg/dL)	AA	72	47.50 (43.00-57.00)	Mann-Whitney	0.0738
	AG	27	56.00 (45.00-63.00)		
	GG	0	-	-	
Triglycerides (TG)	AA	72	140.50 (113.00-206.75)	Mann-Whitney	0.4083
	AG	27	159.00 (108.00-250.00)		
	GG	0	-	-	
HSI	AA	72	45.00 (41.03-48.73)	Mann-Whitney	0.4518
	AG	27	47.00 (40.00-51.60)		
	GG	0	-	-	
FLI	AA	72	91.14 (74.29-96.75)	Mann-Whitney	0.8422
	AG	27	90.68 (76.94-96.02)		
	GG	0	-	-	

Data are presented as mean ± SD or median (IQR), as appropriate. Normality of distribution was assessed using the Shapiro-Wilk test. Comparisons between genotype groups were performed using Kruskal-Wallis test, or Mann-Whitney U test, depending on number of analyzed groups. Statistical significance was set at $p<0.05$.

Supplementary Table S7. Genotype-phenotype associations for *XRCC1* rs25487 polymorphism in the NAFLD group.

Clinical/biochemical parameter	Genotype	n	Mean ± SD/ Median (IQR)	Test used	p-value
BMI (kg/m²)	CC	39	32.72 (28.76- 36.14)	Kruskal-Wallis	0.4400
	CT	46	32.48 (27.94- 37.02)		
	TT	14	33.83 (31.59- 38.46)		
HDL (mg/dL)	CC	39	48.00 (44.00-57.00)	Kruskal-Wallis	0.7209
	CT	46	49.00 (44.75-60.00)		
	TT	14	51.00 (43.75-59.00)		
Triglycerides (TG)	CC	39	137.00 (108.00-187.00)	Kruskal-Wallis	0.1936
	CT	46	159.00 (119.75-230.00)		
	TT	14	126.50 (100.75-203.00)		
HSI	CC	39	45.30 (41.40- 49.85)	Kruskal-Wallis	0.8290
	CT	46	45.15 (39.90- 50.60)		
	TT	14	44.85 (43.20- 49.60)		
FLI	CC	39	90.93 (75.13-94.85)	Kruskal-Wallis	0.7940
	CT	46	91.11 (73.73-96.80)		
	TT	14	91.62 (77.11-96.68)		

Data are presented as mean ± SD or median (IQR), as appropriate. Normality of distribution was assessed using the Shapiro-Wilk test. Comparisons between genotype groups were performed using Kruskal-Wallis test, or Mann-Whitney U test, depending on number of analyzed groups. Statistical significance was set at $p<0.05$.

Supplementary Table S8. Genotype-phenotype associations for *XRCC1* rs1799782 polymorphism in the NAFLD group.

Clinical/biochemical parameter	Genotype	n	Mean ± SD/ Median (IQR)	Test used	p-value
BMI (kg/m²)	AA	21	34.04 (29.78-36.39)	Kruskal-Wallis	0.5565
	AG	9	30.12 (27.58-35.75)		
	GG	69	32.72 (28.75-37.03)		
HDL (mg/dL)	AA	21	47.00 (44.00-56.50)	Kruskal-Wallis	0.7076
	AG	9	57.00 (41.00-62.50)		
	GG	69	49.00 (43.50-58.50)		
Triglycerides (TG)	AA	21	132.00 (100.50-208.50)	Kruskal-Wallis	0.4854
	AG	9	145.00 (114.00-200.00)		
	GG	69	150.00 (116.00-211.50)		
HSI	AA	21	44.90 (42.38- 49.63)	Kruskal-Wallis	0.5690
	AG	9	42.00 (38.90- 48.00)		
	GG	69	46.00 (40.65- 50.08)		
FLI	AA	21	90.55 (77.01-96.06)	Kruskal-Wallis	0.6803
	AG	9	76.94 (67.31-96.57)		
	GG	69	91.5 (76.36-96.74)		

Data are presented as mean ± SD or median (IQR), as appropriate. Normality of distribution was assessed using the Shapiro-Wilk test. Comparisons between genotype groups were performed using Kruskal-Wallis test, or Mann-Whitney U test, depending on number of analyzed groups. Statistical significance was set at $p<0.05$.
